# Uncovering differences in cadmium accumulation capacity of different *Ipomoea aquatica* cultivars at the level of root cell types

**DOI:** 10.1093/hr/uhaf077

**Published:** 2025-03-11

**Authors:** Chuang Shen, Bai-Fei Huang, Qiong Liao, Kai-Feng Chen, Jun-Liang Xin, Ying-Ying Huang

**Affiliations:** Research Center for Environmental Pollution Control Technology, School of Chemical and Environmental Engineering, Hunan Institute of Technology, Heng Hua Road 18, Hengyang 421002, China; Research Center for Environmental Pollution Control Technology, School of Chemical and Environmental Engineering, Hunan Institute of Technology, Heng Hua Road 18, Hengyang 421002, China; Hunan Chemical Vocational Technology College, Wisdom Road 118, Zhuzhou 412000, China; Research Center for Environmental Pollution Control Technology, School of Chemical and Environmental Engineering, Hunan Institute of Technology, Heng Hua Road 18, Hengyang 421002, China; Research Center for Environmental Pollution Control Technology, School of Chemical and Environmental Engineering, Hunan Institute of Technology, Heng Hua Road 18, Hengyang 421002, China; Research Center for Environmental Pollution Control Technology, School of Chemical and Environmental Engineering, Hunan Institute of Technology, Heng Hua Road 18, Hengyang 421002, China

## Abstract

Water spinach (*Ipomoea aquatica*) can accumulate cadmium (Cd) even in mildly contaminated soils, but the roles of its root tip cell types in Cd fixation and transport remain unclear. Single-cell RNA sequencing revealed nine cell types in root tips in both the QLQ cultivar (low Cd accumulation) and the T308 cultivar (high Cd accumulation). High expression of *LAC2* and *PER72* in the QLQ epidermis was associated with enhanced lignin deposition, which may facilitate fixation of Cd and reduce its translocation to the shoot. In T308, *PER72* and hormone-related genes (*PIN1*, *ARF8*, *IAA17*, and *EIN3*) were upregulated, which was hypothesized to promote xylem and trichoblast development, potentially facilitating Cd uptake and transport. Fluorescence assays suggested that the higher pectin demethylation and lignin content in QLQ may limit Cd movement, whereas the more developed tissues in T308 may contribute to increased Cd accumulation in the shoots. These findings clarify the mechanisms by which Cd accumulates in water spinach and offer insights into mitigating Cd uptake in crops.

## Introduction

Cadmium (Cd) pollution is a global issue posing significant threats to human health via the food chain [1]. The bioavailability and phytotoxicity of Cd in soil–plant systems are intrinsically linked to its chemical speciation. In contaminated soils, Cd exists primarily as Cd^2+^ ions and complexes with inorganic ligands (e.g. CdCl^+^, CdSO₄) or organic chelates (e.g. Cd-humate), with ionic Cd^2+^ being the form that is most bioavailable for plant uptake [2]. The rhizosphere microenvironment critically modulates Cd speciation through pH fluctuations and root exudates. Acidic conditions (pH <6.0) elevate the solubility of Cd by promoting the dissociation of Cd-carbonates and Cd-oxides, while alkaline conditions (pH >7.5) favor the precipitation of Cd as Cd(OH)₂ or Cd₃(PO₄)₂ [3]. At the cellular level, the cell wall, composed of cellulose, hemicellulose, and pectin, contains functional groups like carboxyl, hydroxyl, and thiol, which play crucial roles in binding Cd^2+^, thereby reducing Cd uptake into cells [4]. When Cd enters the cell, the functional thiol groups in glutathione (GSH), phytochelatins (PCs), metallothioneins (MTs), and organic acids exhibit a strong affinity for it, chelating Cd ions to neutralize their toxicity and facilitate their sequestration in plant vacuoles [5].

*Ipomoea aquatica* (water spinach), a vegetable that is widely consumed in Southeast Asia and around the world, takes up and accumulates large amounts of Cd even when grown in only slightly contaminated soil. To address this issue, the concept of Cd pollution-safe cultivars (PSCs) has been proposed, focusing on the screening and breeding of crop cultivars that accumulate little Cd accumulation even in contaminated soils [6]. Our previous comparative physiological and molecular studies of water spinach revealed that the low-Cd-accumulating cultivar QLQ has a thicker root epidermal cell wall and higher lignin content than the high-Cd-accumulating cultivar T308 [7]. The total amount of pectin and the percentage of demethylated pectin are the two main factors affecting pectin’s ability to fix Cd. In rice, the increased levels of pectin demethylation and total pectin concentration significantly enhance Cd fixation [8]. Our previous study revealed that the level of pectin methylation had a greater effect on the fixation of Cd in the cell wall of hot pepper leaves than did the total pectin concentration [9]. Additionally, studies on the Cd-tolerant *Glycine max* (soybean) cultivar HX3 found an association between higher Cd fixation in the cell wall and increased pectin methylesterase (PME), as well as a higher proportion of demethylated pectin [10]. Higher lignin content and lower pectin methylesterification (regulated by PME) levels are more effective at preventing Cd entry into cells and reducing its transport to the shoots [11]. It has also been demonstrated that a greater number of root hairs helps to promote transpiration, which is the primary force driving the upward transport of Cd to the above-ground parts [12]. However, roots are composed of tissues such as root cap, epidermis, endodermis, and trichoblast (root hairs) [13], and whether the cell wall components of these tissues differ in their function regarding Cd fixation, uptake, and transport requires further investigation.

The root apical meristem generates various root cell types through processes such as cell differentiation and division [14]. These root structures significantly increase the root’s surface area in contact with the environment, enhancing Cd uptake, underscoring its essential function in the accumulation of Cd by plants. The Cd concentration in *Oryza sativa* (rice) root tips was found to be 1.4-fold higher than that in other root areas under Cd treatment [15]. Quantitative analyses of gene expression in specific cell types are essential for understanding the complex genetic regulatory networks that determine root development in response to biotic and abiotic stresses [16]. Plant hormones such as abscisic acid (ABA) and auxin play crucial roles in regulating root development and stress responses under Cd stress, as observed in plants like rice and *Arabidopsis thaliana* [17]. Xylem development is crucial because it serves as the main conduit for Cd transport to the plant’s aerial parts, influencing the levels of accumulation of Cd [18]. However, the influence of plant hormones on the differential development and hierarchical reconstruction of the xylem and root hairs in the root tips of water spinach cultivars with varying levels of Cd accumulation remains unclear.

Unlike traditional sequencing, which focuses on whole tissues, single-cell RNA sequencing (scRNA-seq) allows researchers to identify distinct cell types within plant tissues and analyze their unique gene expression profiles [19]. Advances in single-cell RNA sequencing (scRNA-seq) have revealed the diversity of cell types, lineage relationships, and gene expression patterns in plant tissues (e.g. roots, embryos, and leaves) of *Arabidopsis thaliana* and *Fragaria vesca*, highlighting a high degree of heterogeneity of these tissues and an unprecedented range of expression profiles of major cell types [20, 21]. Wang *et al.* [22] used scRNA-seq in rice to reveal the heterogeneity of cell types in response to abiotic stress. Meanwhile, using scRNA-seq, Wendrich *et al.* [23] discovered that cytokinin signaling in *Arabidopsis thaliana* root vascular cells might play a role in root hair responses to low phosphate levels. However, relatively few scRNA-seq studies have been conducted on plant responses under Cd stress, let alone comparisons between cultivars that accumulate Cd at different levels. 

In our previous study, we found that the Cd content in the shoots of water spinach T308 was 3.1 to 3.4 times higher than that of QLQ, while the Cd content in the cell walls of QLQ roots was 1.5 to 1.8 times higher than that of T308 [24]. This indicates that the adsorption and fixation of Cd by the root cell walls determine the Cd content in the shoots to some extent. However, our previous studies on the differences in Cd accumulation between water spinach cultivars focused mainly on the whole tissue, which obscured the specific roles of individual cell types in Cd uptake, transport, and fixation. Because the root system plays a much larger role in Cd absorption and transport than does aerial tissues, we analyzed the differences in the response mechanisms of root cells in QLQ and T308 under Cd treatment using scRNA-seq and reported the gene expression profiles of the major cell types in the root tips. In addition, through microscopic observation, lignin content measurement, and analysis of PME activity, we identified the cell types responsible for the differences in ability to accumulate Cd. This study not only reveals the mechanisms underlying the differences in Cd uptake and accumulation between different crop cultivars at the scRNA-seq level but also provides new insights and theoretical support for screening Cd-PSC.

## Results

### Identification of cell types in water spinach root tips upon Cd exposure via scRNA-seq

When the seedlings of water spinach developed two true leaves, they were transplanted into a Cd-free 1/2 Hoagland nutrient solution and allowed to acclimate for 3 days. The seedlings were then cultured in a 1/2 Hoagland nutrient solution containing 0.5 mg/L of Cd for 7 days, and the root samples were collected for scRNA-seq analysis. The gene expression matrix of QLQ included 11 815 total detected genes in 19 705 filtered cells and yielded an average unique molecular identifier (UMI) of 1426 per cell (Table S1). With T308, the gene expression matrix included 11 831 total detected genes in 21 603 filtered cells with an average UMI of 1327 per cell (Table S1). The overall normalized expression levels from scRNA-seq showed strong correlations with the bulk RNA-seq data for QLQ (Fig. S1A) and T308 (Fig. S1B), with Pearson’s correlation coefficients of 0.80 and 0.83, respectively. This indicated that scRNA-seq reliably represents the transcriptomic response of the root tips to Cd stress.

**Figure 1:**
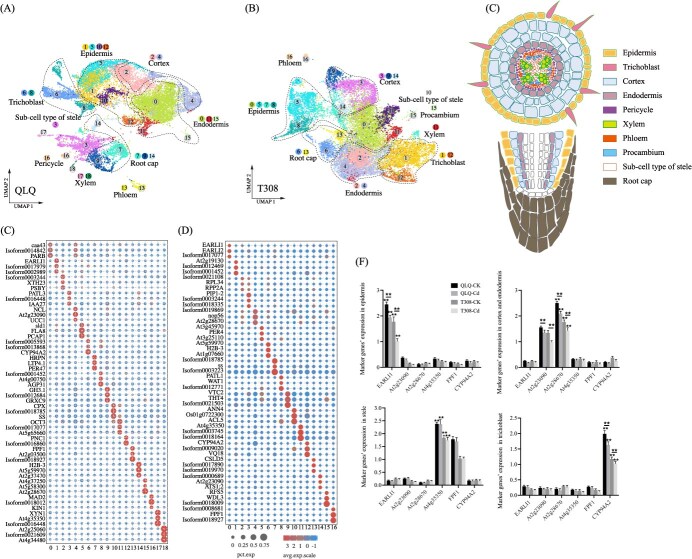
scRNA-seq analysis and cell type identification in QLQ and T308 root tip Cells. (A, B) UMAP visualization of cell clusters and cell types in QLQ and T308. Each dot represents a single cell, with colors indicating cell clusters or cell types. (C) Schematic of anatomy of water spinach root tip. (D, E) Expression patterns of marker genes in QLQ and T308. (F) qPCR validation of the key marker genes of different cell types. Notes: Values are the mean ± standard error (*n* = 3). Statistical significance was determined using ANOVA with LSD, and ‘**’ indicates significant differences (*P* < 0.01) under different treatments.

To uncover the local similarity and global structure of the cell populations, the Uniform Manifold Approximation and Projection (UMAP) algorithm was employed and 19 clusters for QLQ and 17 clusters for T308 root tip cells were obtained (Fig. 1A and B). To classify the clusters into specific cell types, we first identified cluster-enriched genes and specific marker genes for each cluster in QLQ (Table S2) and T308 (Table S3). Subsequently, these genes were compared with known marker genes published in the PlantCellMarker database (https://www.tobaccodb.org/pcmdb/), primarily derived from *Arabidopsis thaliana* and rice, to accurately assign each cluster to its corresponding cell type. The cross-sectional image of the root tip tissue of water spinach illustrates the distribution and arrangement of various cell types (Fig. 1C). The marker genes for each cluster of QLQ and T308 are shown in Fig. 1D and E, respectively, with the comparison of marker genes detailed in Tables S3 and S4. Since water spinach is a non-model plant, we only separated the clusters of xylem, phloem, procambium and pericycle from stele based on marker genes, and the other stele cells that could not be specified were classified as sub-cell type of stele. Finally, the clusters of QLQ root tip cells were classified into nine cell types: epidermis (clusters 1, 5, 10, 12), cortex (clusters 2, 4), endodermis (clusters 0, 11, 15), root cap (clusters 7, 9, 14), sub-cell type of stele (cluster 3), trichoblast (clusters 6, 8), phloem (cluster 13), pericycle (cluster 16), and xylem (clusters 17, 18) (Fig. 1A). Similarly, the clusters in T308 were categorized into nine cell types: epidermis (clusters 0, 5, 7, 8), endodermis (clusters 2, 4), trichoblast (clusters 1, 12), cortex (clusters 3, 9, 14), root cap (clusters 6, 13), sub-cell type of stele (cluster 10), xylem (cluster 11), procambium (cluster 15), and phloem (cluster 16) (Fig. 1B). To validate the tissue types obtained by scRNA-seq, the epidermis, cortex, endodermis, stele, and root hair tissues were dissected from the root tips of QLQ and T308 cultivars under both CK (7 d in nutrient solution without Cd) and Cd treatments using a dissection microscope, tweezers, and scissors (Fig. S2). Thereafter, we performed quantitative (qPCR) to confirm the expression levels of marker genes associated with the epidermis, cortex and endodermis, stele, and trichoblast. The results were consistent with those from scRNA-seq, confirming the accuracy of the cell type classification (Fig. 1F). Interestingly, the expression levels of marker genes for the epidermis, cortex and endodermis, and trichoblast under CK treatment were significantly higher than those under Cd treatment (Fig. 1F), indicating that Cd exposure exerts an inhibitory effect on the development of the epidermis, cortex and endodermis, and trichoblast.

When comparing QLQ to T308, the number of more highly expressed DEGs in the epidermis, cortex, and sub-cell type of stele was significantly higher than the number of DEGs with lower expression, whereas the opposite was true in the trichoblast and xylem (Fig. S3A). To investigate the function of DEGs in each cell type of QLQ and T308 under Cd stress, we performed Gene Ontology (GO) analysis. GO terms such as cellular process, metabolic process, and cell part were significantly enriched in the sub-cell type of stele, cortex, and trichoblast, while GO terms such as response to stimulus, and transporter activity were highly enriched in the phloem (Fig. S3B). The differences in GO terms indicate that the cell types in QLQ and T308 exhibited different characteristics under Cd stress.

### Developmental trajectories of xylem and analysis of lignin content

**Figure 2:**
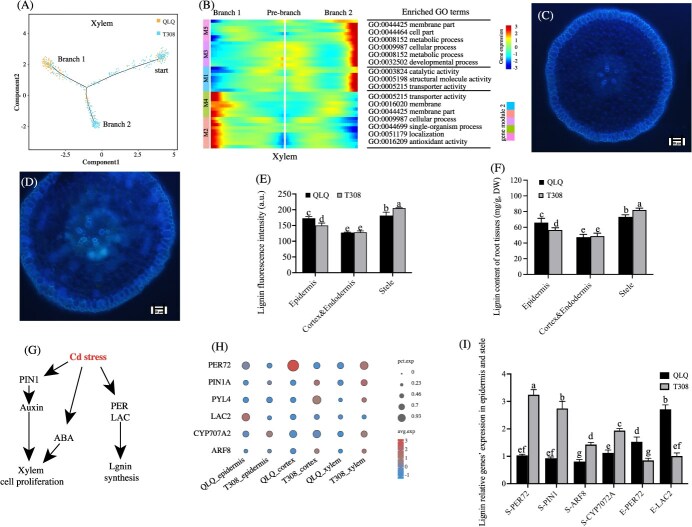
Comparison of xylem development and lignin content in the root tips of QLQ and T308. (A) Pseudotime analysis of xylem development. (B) Heatmap showing gene expression patterns during xylem differentiation. (C, D) Cross-sections of root tips of QLQ and T308 under UV for lignin fluorescence analysis. (E) Lignin fluorescence intensity of root tips of QLQ and T308. (F) Lignin content of root tip tissues. (G, H, I) Gene expression profiles related to xylem development and lignin biosynthesis pathways under Cd stress (G), and their scRNA-seq results (H) and qPCR validation results (I) of their expression in different cell types. Notes: Values are the mean ± standard error (*n* = 3). Statistical significance was determined using ANOVA with LSD, and different small letters indicate significant differences (*P* < 0.05) under different treatments.

The xylem serves as the primary conduit for transporting water and nutrients (including Cd) in plants. To better understand the genetic regulation underlying xylem differentiation, we reconstructed the developmental trajectories of xylem cells. Our analysis revealed distinct developmental pathways for xylem cells in QLQ and T308: QLQ cells primarily progressed toward branch 1, while T308 cells were concentrated in branch 2 (Fig. 2A). Xylem cells were categorized into five clusters by colored pseudotime trajectory analysis as follows. Cluster M1, associated with prebranch cells, was enriched for GO terms related to catalytic activity (GO:0003821), structural molecule activity (GO:0005198), and transporter activity (GO:0005215). Clusters M3 and M5, corresponding to branch 1 cells, were primarily involved in the membrane part (GO:0044425), cell part (GO:0044464), metabolic processes (GO:0008152), and cellular processes (GO:0009987). Clusters M4 and M5, linked to branch 2 cells, showed functions related to transporter activity (GO:0005215), membrane (GO:0016020), membrane part (GO:0041425), cellular processes (GO:0009987), single organism process (GO:0044699), localization (GO:0051179), antioxidant activity (GO:0016209), and developmental processes (GO:0032502) (Fig. 2B).

Lignin fluorescence staining of the roots indicated that QLQ exhibited higher lignin fluorescence intensity in the epidermis than did T308, while QLQ had markedly lower lignin fluorescence in the stele, especially in the xylem (Fig. 2C–E), than did T308. Additionally, measurements of lignin content in different root tissues revealed that QLQ and T308 had lignin contents of 73.23 and 82.05 mg/g (dry weight, DW) in the stele, respectively, which were significantly higher than the levels of 65.95 and 56.58 mg/g in the epidermis (Fig. 2F). Lignin content in the cortex and endodermis was the lowest. Overall, these findings demonstrate that the lignin content in QLQ and T308 correlates well with the observed lignin fluorescence results.

Additionally, based on the pathways regulating xylem development and lignin content [23], several key genes were selected to analyze their expression level (Fig. 2G). The scRNA-seq results (Fig. 2H) of these gene expression levels were consistent with the qPCR validation (Fig. 2I), and their expression levels showed significant differences between QLQ and T308 (*P* < 0.05). Specifically, xylem development-related genes such as *PIN FORMED1* (*PIN1*), *AUXIN RESPONSE FACTORS* (*ARF8*), and *ABA 8′-hydroxylase* (*CYP707A2*) were expressed at notably higher levels in the xylem of T308 than in QLQ (Fig. 2H and I). Conversely, *LACCASE2* (*LAC2*) and *PEROXIDASE 72* (*PER72*) showed significantly higher expression levels in the epidermis of QLQ than in T308. However, *PER72* expression was significantly lower in the xylem of QLQ than in T308 (Fig. 2H and I). These differential expression levels of the key genes may contribute to the observed differences in lignin content and xylem development between QLQ and T308.

### Developmental trajectories of trichoblast

**Figure 3:**
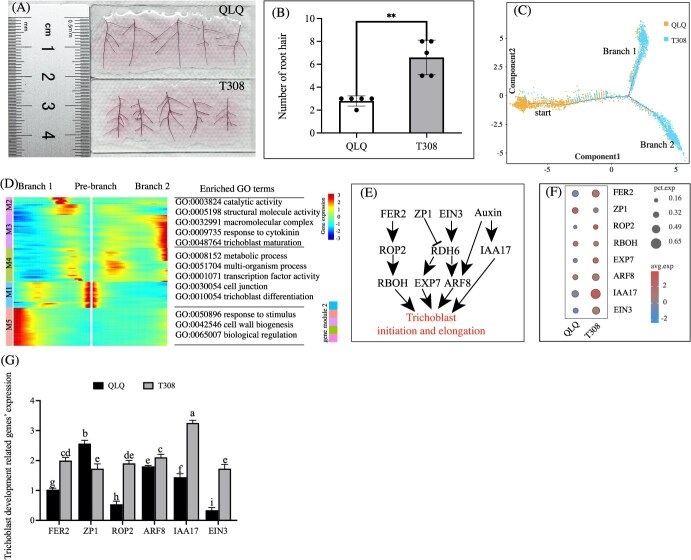
Comparison of trichoblast development between QLQ and T308. (A) Root hair status of the root tip zone of QLQ and T308. (B) Number of root hair of the root tip zone of QLQ and T308. (C) Pseudotime analysis of trichoblast development. (D) Heatmap showing gene expression patterns during trichoblast differentiation. (E, F, G) The regulatory pathways and expression profiles of trichoblast development-related genes under Cd stress. Notes: Values are presented as the mean ± standard error (*n* = 3). Statistical significance was determined using ANOVA with LSD, and different small letters and ‘**’ indicate significant differences (*P* < 0.05 and *P* < 0.01) under different treatments.

Photographs taken 1.0–2.0 cm from the root tip showed that the number of root hairs was significantly lower in QLQ than in T308 (Fig. 3A and B). Since root hair is initiated from the trichoblast, this suggests that the two cultivars differed in terms of trichoblast development. To explore this further, we conducted pseudotime trajectory analysis on trichoblast cells from both cultivars. The results revealed that QLQ trichoblast cells were primarily at earlier developmental stages, while T308 trichoblast cells appeared to branch out, with notable enrichment in differentiation branches 1 and 2 (Fig. 3C). This suggests that the trichoblast cells from QLQ and T308 showed divergent differentiation and developmental features.

**Figure 4:**
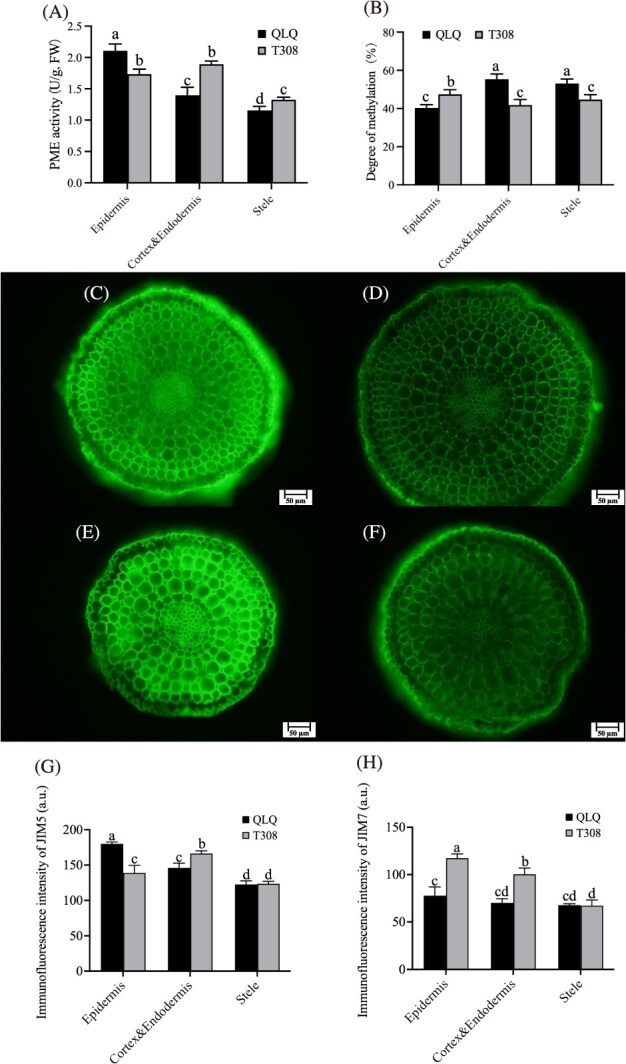
Analysis of Pectin and Cd Content in the root of QLQ and T308. (A) PME activity in root tissues. (B) Degree of pectin methylation. (C, D) Immunofluorescence of JIM5 and JIM7 of QLQ root tips. (E, F) Immunofluorescence of JIM5 and JIM7 of T308 root tips. (G, H) JIM5 and JIM7 fluorescence intensity of root tips sections of QLQ and T308. Notes: Values are the mean ± standard error (*n* = 3). Statistical significance was determined using ANOVA with LSD, and different small letters indicate significant differences (*P* < 0.05) under different treatments.

Pseudotime trajectory analysis, supplemented by heatmap visualization, categorized trichoblast cells into five groups (M1–M5) (Fig. 3D). M1 and M4 represent prebranch cells; these groups are involved in metabolic processes (GO:0008152), multi-organism processes (GO:0051704), transcription factor activity (GO:0001071), cell junctions (GO:0030054), and trichoblast differentiation (GO:0010054). M5 cells correspond to branch 1, which is involved in functions related to response to stimuli (GO:0050896), cell wall biogenesis (GO:0042546), and biological regulation (GO:0065007). M2 and M3 represent cells of branch 2, and these groups are characterized by catalytic activity (GO:0003824), structural molecule activity (GO:0005198), macromolecular complexes (GO:0032991), response to cytokinin (GO:0009735), and trichoblast maturation (GO:0048764).

In addition, we identified several key genes associated with trichoblast development based on the regulatory pathway of root hair development [25] and their expression levels in scRNA-seq in this study (Fig. 3E). The scRNA-seq results (Fig. 3F) of these gene expression levels were also consistent with the qPCR validation results (Fig. 3G). *Feronia2* (*FER2*) and *Rho of Plants 2* (*ROP2*) were significantly more expressed in the T308 trichoblast than in QLQ (Fig. 3F and G). Conversely, the transcription factor-related gene *zinc finger protein 1* (*ZP1*) exhibited higher expression in the QLQ trichoblast (Fig. 3F and G). Hormone-related genes, including *ARF8*, *auxin co-receptor* (*IAA17*), and *ethylene-insensitive 3* (*EIN3*), were also more highly expressed in the T308 trichoblast than in QLQ (Fig. 3F and G). These differences in gene expression likely contributed to the observed disparities in trichoblast development between QLQ and T308.

### Analysis of the PME activity and pectin methylesterification levels

The assessment of root PME activity revealed that, in QLQ, the epidermis had the highest PME activity, followed by the cortex and endodermis, and the stele had the lowest PME activity. Conversely, T308 exhibited similar PME activity levels in the epidermis and endodermis, both of which were significantly higher than in the stele. Moreover, the PME activity in the QLQ epidermis was substantially higher than that in T308, while it was markedly lower in the QLQ cortex and endodermis and stele than for T308 (Fig. 4A). In QLQ, the cortex, endodermis, and stele exhibited the highest levels of pectin methylesterification, whereas in T308, these tissues exhibited lower levels of pectin methylesterification than the epidermis. Notably, QLQ displayed significantly lower pectin methylesterification in the epidermis than T308 did, whereas the cortex, endodermis, and stele in QLQ showed significantly higher levels of methylesterification than in T308 (Fig. 4B). Overall, there was a negative correlation between PME activity and the degree of pectin methylesterification.

JIM5 and JIM7 indicate the degrees of pectin demethylation and methylation, respectively. Immunofluorescence staining of pectin showed that the fluorescence intensity of JIM5 was significantly higher in the QLQ epidermis than in T308 (*P* < 0.05), while it was significantly lower in the QLQ cortex and endodermis than in T308 (Fig. 4C, E, and G). In contrast, the fluorescence intensity of JIM7, which reflects pectin methylesterification, was significantly higher in the epidermis, cortex, and endodermis of T308 than in QLQ (*P* < 0.05) (Fig. 4D, F, and H).

### Analysis of the Cd content in root tissues

**Figure 5:**
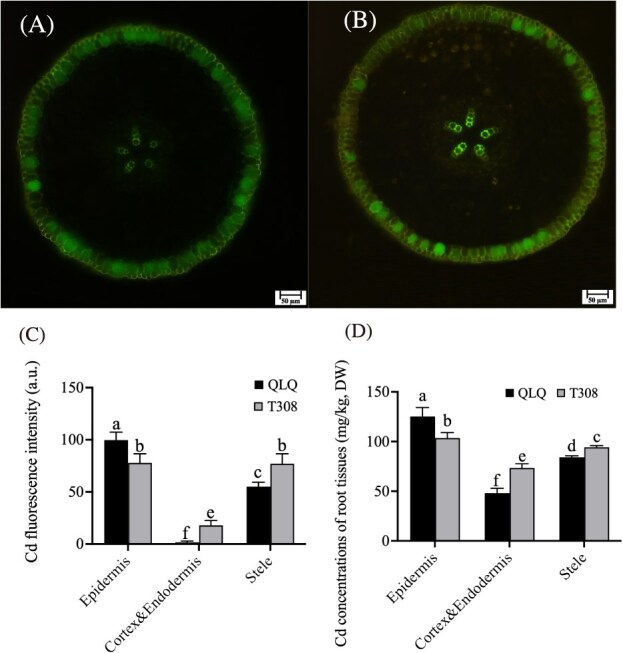
Cd Content and fluorescence analysis of QLQ and T308 root tips. (A) Cd fluorescence analysis of QLQ root tip sections. (B) Cd fluorescence analysis of T308 root tip sections. (C) Cd fluorescence intensity analysis of QLQ and T308 root tip sections. (D) Root tip Cd content of the different cell types of QLQ and T308. Notes: Values are the mean ± standard error (*n* = 3). Statistical significance was determined using ANOVA with LSD, and different small letters indicate significant differences (*P* < 0.05) under different treatments.

Leadmium™ Green AM staining was used to evaluate the Cd distribution in the root tissues of QLQ and T308. By observing Cd fluorescence, it was found that, in the epidermis of QLQ and T308, Cd fluorescence was present both in the cell walls and inside the cells, suggesting that Cd may be sequestered in both of these locations (Fig. 5A and B). In the cortex and endodermis, only a few T308 cells emitted slight fluorescence, while QLQ showed almost no fluorescence at all (Fig. 5A and B). Notably, the Cd fluorescence intensity in the stele of T308 was significantly greater than that in the stele of QLQ, whereas the Cd fluorescence intensity showed the opposite pattern in the epidermis (Fig. 5C). Additionally, the spatial distribution of Cd fluorescence in both QLQ and T308 corresponded with the regions of lignin fluorescence.

FAAS measurements indicated that the Cd content reached its highest level in the QLQ epidermis, at 125.25 mg/kg, which was significantly greater than the 103.50 mg/kg observed in T308 (Fig. 5D). Both QLQ and T308 had significantly lower Cd content in the cortex and endodermis than in the epidermis and stele, with QLQ showing significantly lower Cd content in the stele compared with T308 (Fig. 5D). Conversely, the Cd content in the stele was significantly higher in T308 than in QLQ.

## Discussion

### Accurately distinguishing the major root cell types in water spinach using scRNA-seq

Analysis of the molecular and physiological mechanisms of low Cd accumulation in crops facilitates the screening and cultivation of Cd-PSCs. In our previous studies, the transcriptomic and proteomic differences between the high-Cd-accumulating water spinach cultivar T308 and the low-Cd-accumulating cultivar QLQ were analyzed at the level of the total root system, and their effects on Cd uptake and transport were investigated [7, 26]. types affect Cd uptake, accumulation, and transport has not been comprehensively studied. Using scRNA-seq, we identified nine cell types in the root tips of both QLQ and T308. Interestingly, the only notable difference was that pericycle cell types were exclusively identified in QLQ, whereas procambium cell types were found only in T308. Because water spinach is a nonmodel plant, we could only distinguish the cell types within the stele using marker genes from known species. Similarly, the single-cell studies on woodland strawberry and *Arabidopsis thaliana* by Bai *et al.* [20] and Zhang *et al.* [13], respectively, also encountered difficulties in accurately identifying the cell types for each cluster. Therefore, the unidentified procambium in QLQ and pericycle in T308 in this study are likely included within the respective sub-cell types of the stele. Additionally, we manually isolated the epidermis, cortex and endodermis, stele, and trichoblast tissues from the root tips of water spinach to perform qPCR validation of the corresponding marker genes identified by scRNA-seq, ensuring the accuracy of the scRNA-seq results. Although this method is less intuitive than in situ hybridization, it is equally reliable for verifying the results. Similar approaches have been employed by An *et al.* [27] and Liang *et al.* [28] to accurately validate scRNA-seq findings in *Hevea brasiliensis* (rubber tree) and *Populus* L. (poplar), respectively.

### Spatial distribution and role of lignin in Cd fixation in water spinach roots

Our previous transcriptomic and proteomic studies revealed that the expression levels of lignin synthesis-regulating genes, *LACs*, were significantly higher in the roots of QLQ than in T308, leading to higher Cd content in QLQ root cell walls and lower Cd accumulation in the shoots in comparison to the levels in T308 [7, 26]. Both LAC and PER are key enzymes involved in lignin synthesis and play important roles in secondary cell wall formation in plants [29]. The scRNA-seq results of this study revealed that *LAC2* and *PER72* were expressed at significantly higher levels in the epidermis and cortex of QLQ than in T308, whereas *PER72* was expressed at significantly lower levels in the xylem of QLQ than in T308. This suggests that the spatial distribution of the expression if lignin synthesis-regulating genes differed between the two cultivars. Supporting this, histochemical analysis of *Medicago truncatula* showed that Cd exposure induced intense lignification in the epidermis and xylem of the roots [30]. Consistent with these findings, lignin fluorescence and content analysis in the present study indicated higher lignin content in the epidermis of QLQ, while the xylem lignification was more pronounced in T308, further confirming the tissue-specific variation in lignin deposition. Lignin has a strong affinity for Cd due to its side-chain structure, and studies have demonstrated that high lignin content in cell walls helps fix Cd, preventing it from entering the cells and thereby enhancing the plant’s Cd tolerance [31]. Moreover, lignin has been shown to reduce Cd translocation to the shoots, effectively decreasing the Cd content in rice grains [32]. The findings of the present study demonstrated that Cd fluorescence coincided with the location and intensity of lignin fluorescence in the epidermis of water spinach, aligning with the findings in these previous reports. This suggests that lignin content plays a key role in the variation of Cd fixation capacity in the root epidermis among different water spinach cultivars.

### Auxin and ABA regulation of xylem development facilitates Cd transport in T308

Plant hormones such as ABA and auxin play critical roles in root xylem development. In *Arabidopsis thaliana*, ABA promotes root xylem development by repressing the expression of the transcription factor *HD-ZIPIII* [33], while auxin regulates the differentiation of root xylem through activation of the vascular-related NAC domain (VND7) [34]. Furthermore, ARF8 interacts physically with DELLA proteins to specifically inhibit phloem proliferation and induce phloem senescence during the expansion phase of xylem [35]. In this study, the expression of auxin-regulated genes such as *PIN1* and *ARF8* and genes for ABA receptors such as *PYL4* and *CYP707A2* was significantly increased in T308 xylem compared with the levels in QLQ. This upregulation likely contributes to the enhanced xylem development observed in T308. Xylem exhibits a high affinity for Cd, and the Cd content in xylem sap determines its content in the plant shoots [36]. Under Cd stress, plants may reduce Cd transport to the shoots by reducing the size and number of xylem vessels in the roots [37]. Compared with the Cd-non-hyperaccumulating ecotype of *Sedum alfredii*, the Cd-hyperaccumulating one possessed more and larger xylem vessels, which was a key contributor to its high Cd accumulation [38]. Thus, in this study, the well-developed xylem in T308 should facilitate Cd transportation, which could be the main reason for its high Cd accumulation in the shoot.

### Trichoblast development and hormonal regulation facilitate Cd uptake in T308

Non-essential toxic elements such as Cd can also be absorbed by root hairs through various metal transporters [39]. Trichoblasts develop in specific regions of the root epidermis and differentiate into root hairs, enlarging the contact area between the roots and the environment, and their length and number are often related to plants’ uptake and accumulation of Cd [40]. In this study, the root tips of T308 exhibited more root hairs than those of QLQ, which can be attributed to the enhanced development of trichoblasts in T308. Pseudotime analysis further showed that the degree of trichoblast differentiation of T308 was higher than that of QLQ, in accordance with the results on the root hair status. To explore the molecular mechanisms behind root hair development in T308, focus was placed on the key genes regulating trichoblast development. Feronia (FER) plays an important role in trichoblast development and environmental stress response; the *fer-4* mutant showed an approximately 50% reduction in the Cd concentration in the roots [41]. ROP2 is a positive regulator of trichoblasts development [42]. The high expression levels of *FER2* and *ROP2* in T308 trichoblasts cell clusters likely promote the initiation and development of trichoblasts. In addition, hormone-related genes such as *ARF8*, *IAA17*, and *EIN3*, which promote root hair growth [43, 44], are expressed at higher levels in T308 trichoblasts than in QLQ. These genes should contribute to the increased number of T308 root hairs. Higher root hair density enhances transpiration, which drives Cd transport to the aerial parts [12]. Therefore, it is reasonable to conclude that trichoblast development-related genes and hormones facilitate trichoblast initiation and promote the development of root hairs in T308. This adaptation enables T308 to absorb larger amounts of Cd, which is subsequently transported upward through transpiration, leading to the greater accumulation of Cd in the shoots.

### Localized pectin demethylation and lignin as key factors in Cd fixation and transport

Pectin is rich in galacturonic acid, which provides numerous negatively charged groups to bind Cd, reducing the likelihood that Cd enters the cell and thereby enhancing the plant’s tolerance to Cd [45]. In the present study, pectin immunofluorescence revealed that, in T308, pectin was predominantly demethylated in the cortex and endodermis, whereas in QLQ, the highest level of pectin demethylation was observed in the epidermis. Combining the findings on the degree of pectin demethylation and Cd content, we can conclude that the activity of pectin in QLQ in the epidermis and T308 in the cortex and endodermis contributed to the Cd fixation in the corresponding parts of these cultivars. This finding refines and updates our previous interpretation of pectin’s role in Cd fixation, shifting from a whole-root perspective to a more localized understanding of pectin’s effects in specific root tissues.

**Figure 6:**
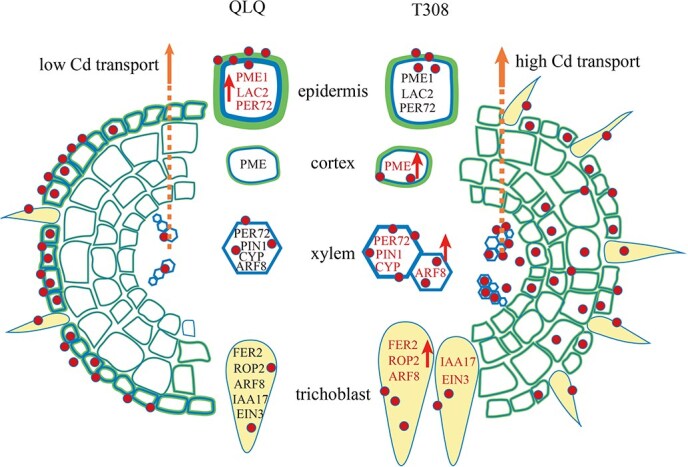
Possible mechanisms of Cd uptake and transport in roots of QLQ and T308. Note: Red text with arrows indicates significantly higher expression levels and blue text denotes significantly lower expression levels (*P* < 0.05).

Another interesting point in this study is that the locations of Cd fluorescence in QLQ and T308 are quite consistent with the lignin fluorescence results, which suggests that lignin is more capable of fixing Cd than pectin is. In roots of maize, 65% of Cd was partially bound to pectin, whereas in *Brassica chinensis*, only 14% of Cd was bound to lignin, which was attributed to the fact that the pectin side chains contained 70% negative charge, resulting in a much higher capacity for Cd fixation than for lignin [31, 46]. In addition, studies have shown that most of the Cd is retained in the root epidermis [47]. The results of Cd fluorescence analysis in the root tips of QLQ and T308 revealed that most of the Cd was fixed in the cell walls or sequestered in the protoplasts of the epidermis, which is in agreement with the results of the above studies. In T308, slight Cd fluorescence appeared inside the cells of the cortex, rather than in the cortical cell walls. In contrast, almost no Cd fluorescence was observed in the cortex of QLQ, making it difficult to conclusively determine the primary pathway for Cd accumulation in the xylem. However, based on Cd fluorescence and Cd content, it is evident that the amount of Cd transported to the xylem in QLQ is significantly lower than that in T308. Slight Cd fluorescence appeared inside the cortex cells of T308 cells rather than in the cell walls, suggesting that environmental Cd mainly enters the xylem via the symplast pathway. In contrast, almost no fluorescence was observed in the endodermis of the QLQ root, making it difficult to determine the primary pathway by which Cd enters the root xylem. However, based on Cd fluorescence and Cd content, it is evident that the amount of Cd entering the xylem of QLQ is significantly lower than that in T308, which should also be an important factor behind the significant difference in their abilities to accumulate Cd in the shoot.

### Conclusion

In summary, through scRNA-seq and a series of subsequent experiments, we performed an in-depth analysis of the mechanisms underlying Cd accumulation in different root tissues of the QLQ and T308 cultivars of water spinach. We also created a diagram to illustrate the potential factors contributing to their differences in Cd fixation and transport (Fig. 6). It is hypothesized that the high expression of *LAC2* and *PER72* in the epidermis of QLQ and the high expression of *PER72* in the xylem of T308 contribute to higher levels of lignin deposition QLQ epidermis and in T308 xylem, respectively. Additionally, the high expression of plant hormone-related genes such as *PIN1*, *ARF8*, *PYL4*, and *CYP707A2* in the xylem of T308 and the high expression of *ARF8*, *IAA17*, and *EIN3* in the trichoblast of T308 could potentially lead to more developed xylem and trichoblasts in T308. The combination of high PME activity, pectin demethylation, and lignin content in the epidermis of QLQ may facilitate Cd fixation, thereby reducing the loading of Cd into the xylem and Cd transport to the shoots. In contrast, the more developed xylem and trichoblast in T308, along with the higher Cd content in the xylem, might promote the transport of Cd to the shoots.

## Materials and methods

### Plant material and treatments

Two water spinach cultivars, T308 and QLQ, were used in this study. When water spinach developed two true leaves, the seedlings were transplanted into Cd-free 1/2 Hoagland nutrient solution and allowed to acclimate for 3 days. Subsequently, the seedlings were divided into two groups: one that continued to grow in the Cd-free 1/2 Hoagland nutrient solution (CK) and another that was cultured in 1/2 Hoagland nutrient solution containing 0.5 mg/L CdCl_2_. After 7 days, root samples from both groups were collected for further analysis. The temperature for cultivation was held between 22 and 28°C with light intensity of 150 μmol·m^−2^·s^−1^, photoperiod of 16 h per day, and average relative humidity of 65%–70%.

### Generating protoplasts

The root tips (1.0–2.0 cm from the apical part of the root) of water spinach treated with 0.5 mg/L Cd were harvested and subjected to digestion at room temperature for 2 h in an enzyme solution devoid of RNase. The digestive solution consisted of 4% cellulase R10, 1.5% microzyme R10, 0.4 M mannitol, 0.1 M MES, 10 mM KCl, 10 mM CaCl_2_, and 0.1% BSA. Digested root tips were filtered three times through a 40 μm cell strainer (Falcon, Cat No./ID: 352340) and washed three times with 8% mannitol at 25°C to obtain protoplasts. The viability of the protoplasts was assessed using the trypan blue exclusion assay to ensure that the viability of each sample exceeded 90%. The concentration of the protoplasts was then adjusted to 1500–2000 cells/μL using a hemocytometer ready for scRNA-seq.

### scRNA-seq and cell clustering

In brief, the suspension of protoplasts was loaded into the 10× Genomics GemCode single-cell instrument to generate single-cell gel bead-in-emulsions (GEMs). scRNA-seq library construction and sequencing were performed using the Chromium Next GEM Single Cell 3′ Reagent Kits v3.1 (10× Genomics, Cat No./ID: P/N 1000075 and 1 000 073) and the Illumina NovaSeq 6000 platform, respectively. The raw scRNA-seq data of this study have been deposited in the China National Center for Bioinformation (BioProject: PRJCA029540). The raw scRNA-seq dataset was originally analyzed using Cell Ranger 3.1.0 (10× Genomics) and gene reads were matched against the water spinach reference genome (https://sra-pub-run-odp.s3.amazonaws.com/sra/SRR10906271/SRR10906271). Low-quality cells and genes were filtered as follows: (i) filtering out genes expressed in one cell or fewer, (ii) retaining the number of genes expressed in more than 500 genes per cell, and (iii) eliminating cells with a proportion of mitochondrial DNA-derived genes greater than 25%. The cell-gene matrix for each sample was imported into Seurat (v.3.1.1), and the high-dimensional cellular data were projected into two-dimensional space using the UMAP (unified mobility approximation and projection) method to cluster cells with similar expression patterns together. Expression values for each gene in a specific cluster were compared with those of other cells using the Wilcoxon rank-sum test. To be defined as differentially expressed genes (DEGs), genes had to be overexpressed at least 1.28-fold in the target cluster and expressed in more than 25% of the cells belonging to the target cluster, along with having a p-value of <0.05. Marker genes were screened using the following criteria: genes were expressed in more than 10% of the cells in a cluster; and mean log2-fold change of upregulated genes >0.59 with *P*-value of <0.01. Marker genes for each cluster were further identified by the FindAllMarkers function in Seurat using a bimodal likelihood ratio test with the default parameters.

### Pseudotime trajectory analysis

Trajectory inference and pseudotime analyses were performed using Monocle2 to select clusters in accordance with the procedure of Trapnell *et al.* [48]. Briefly, the ‘dispersionTable’ and ‘mean_expression’ functions (≥0.1) were first used to identify the genes with high variance. The analysis of dimensional reduction was undertaken using the ‘reduceDimension’ function (max_components = 2, reduction_method = DDRTree). Visualization of trajectories was carried out using ‘plot_cell_trajectory.’ Clustering and plotting of gene expression along branch points were performed using the ‘plot_genes_branched_heatmap’ function.

### Bulk RNA-seq

Total RNA from water spinach root tips, treated with Cd for 7 days, was extracted using the EASYspin Plant RNA Kit (Aidlab, China). The reverse-transcribed cDNA libraries were sequenced using Illumina HiSeq2500 (Illumina, California, USA). After removing low-quality reads, clean reads were used to quantify the expression levels of each cDNA library, measured as fragments per kilobase of transcript per million mapped reads (FPKM). Differentially expressed genes (DEGs) were identified with the criteria of false discovery rate (FDR) <0.05 and |log2 fold change| ≥ 1. Three biological replicates of the bulk RNA-seq libraries were independently generated in parallel. The raw bulk RNA-seq data of this study have been deposited in the China National Center for Bioinformation (BioProject: PRJCA029575). The correlation between the bulk and scRNA-seq datasets was assessed by comparing the average number of reads overlapping each gene between the normalized values of the scRNA-seq and bulk RNA-seq datasets.

### GO and Kyoto Encyclopedia of Genes and Genomes enrichment analyses

GO and Kyoto Encyclopedia of Genes and Genomes (KEGG) enrichment analyses were conducted using the OmicStudio tools (https://www.omicstudio.cn/tool). Significant GO terms and KEGG pathways were defined when the *P*-value was <0.05.

### qPCR validation

An analysis was performed to confirm the cell types and key expressed genes in the root tips of water spinach. Epidermis, cortex and endodermis, stele, and root hair tissues were dissected from the root tips of water spinach cultivars QLQ and T308 under a dissecting microscope using razor blades and tweezers, with three biological replicates collected for each tissue type (Fig. S1). Total RNA was extracted using the MiniBEST Plant RNA Extraction Kit (Takara, Japan), and reverse transcription was performed with the Transcript Uni All-in-One cDNA Synthesis SuperMix (TransGen Biotech, China). qPCR was conducted using ChamQ Universal SYBR qPCR Master Mix (Vazyme, China) on a Bio-Rad CFX Connect Real-Time PCR Detection System. The relative gene expression levels were calculated using the _ΔΔ_Ct method and normalized by the reference gene *IaActin*. Six marker genes were selected to validate tissue identity, while five and six genes were used to assess xylem and trichoblast development, respectively. The qPCR primers used in this study are listed in Table S4.

### Lignin fluorescence and lignin content determination

Briefly, water spinach root tips were hand-sectioned into 20–40-μm-thick slices using a razor blade. The slices were then examined under an Olympus fluorescence microscope (BX35 and U-RFL-T, Japan) with UV light (330–380 nm), where the lignified cell walls exhibited blue fluorescence. Photographs were taken for each sample with consistent exposure time and magnification. For the determination of lignin content, root samples were dissected into the epidermis, endodermis, and stele under a dissecting microscope using a forceps and a scalpel. The lignin content of the root tissues was then measured using a lignin content kit from Suzhou Comin Biotechnology Co., Ltd., following the manufacturer’s instructions.

### Determination of pectin fluorescence, PME activity, and the degree of pectin demethylation

The pectin immunofluorescence of water spinach roots was analyzed using the pectin monoclonal antibodies JIM5 (AS184194; Agrisera, Sweden) and JIM7 (AS184195; Agrisera, Sweden). JIM5 specifically labels low-methylated pectin, whereas JIM7 specifically labels high-methylated pectin. The water spinach root tips were hand-sectioned into 20–40-μm-thick slices using a razor blade. Immunofluorescence of pectin was performed following the method described by Yang *et al.* [49]. Fluorescence imaging was conducted using a fluorescence microscope (BX35 and U-RFL-T; Olympus, Japan), with excitation and emission wavelengths of 488 and 515 nm, respectively. PME activity and the degree of pectin methylation were measured using corresponding kits from Suzhou Comin Biotechnology Co., Ltd., following the manufacturer’s instructions.

### Cd fluorescent staining and determination of Cd in different water spinach root tissues

Leadmium™ Green AM (Molecular Probes, Invitrogen, Carlsbad, CA, USA) was used to investigate the Cd distribution in the roots of the QLQ and T308 cultivars after 7 days of Cd treatment. A stock solution of Leadmium™ Green AM was prepared by adding 50 μL of dimethyl sulfoxide to a vial of dye and then diluting this stock to 500 μL with 0.85% sodium chloride. The water spinach root tips were hand-sectioned into 20–40-μm-thick slices using a razor blade. Root tip slices were incubated with the Leadmium™ Green AM solution in the dark for 90 min. Fluorescence was observed using a fluorescence microscope (BX35 and U-RFL-T; Olympus, Japan). The excitation wavelength of the Cd fluorescence was 484/15 nm, the emission wavelength was 517/30 nm, and the images of the Cd fluorescence were taken at ×100 magnification.

Root samples were dissected into the epidermis, cortex and endodermis, and stele using forceps and a scalpel with the help of a dissecting microscope. Cell wall extraction was performed in accordance with the method of Gao *et al.* [50]. The samples were heat-inactivated at 105°C for 30 min and then dried at 70°C to achieve a constant weight. The dried samples (200 mg) were ground thoroughly and passed through a 60-mesh sieve. These ground samples were then digested with HNO₃ and H₂O₂, following by diluting to 10 mL with deionized water and filtering through a 0.45 μm filter. The Cd concentrations in different plant tissues were measured using flame atomic absorption spectrophotometry (FAAS) (Z-5300; Hitachi, Japan). The quality assurance and quality control (QA/QC) for the experiment included using certified reference materials from plant GBW-07603 (provided by the National Reference Material Center of China).

### Statistical analysis

Quantification of the fluorescence intensity was performed using ImageJ (version 1.54 k), following the method described by Baldacci-Cresp *et al.* [51]. Statistical analyses and figure development were performed using SPSS (version 23.0), R (version 4.4.0), and GraphPad Prism (version 9.0.0). One-way ANOVA with the least significant difference test was employed to determine the significance of the treatment effects. Results were deemed statistically significant at *P* < 0.05.

## Supplementary Material

Web_Material_uhaf077

## Data Availability

The raw scRNA-seq data from this study have been deposited in the China National Center for Bioinformation under BioProject ID: PRJCA029540. Similarly, the raw bulk RNA-seq data are available in the same repository under BioProject ID: PRJCA029575.
