# Supplementary material for: Uncovering differences in cadmium accumulation capacity of different Ipomoea aquatica cultivars at the level of root cell types
Source: Hortic Res. 2025 Mar 11;12(6):uhaf077. doi: 10.1093/hr/uhaf077 (PMC12036322; doi:10.1093/hr/uhaf077)

**Uncovering differences in cadmium accumulation capacity of different *Ipomoea aquatica* cultivars at the level of root cell types**

Chuang Shen^1^, Bai-Fei Huang^1^, Qiong Liao^3^, Kai-Feng Chen^1^, Jun-Liang Xin^1^, Ying-Ying Huang^1^*

^1^ Research Center for Environmental Pollution Control Technology, School of Chemical and Environmental Engineering, Hunan Institute of Technology, Hengyang 421002, China.

^2^ Hunan chemical vocational technology college, Zhuzhou 412000, China.

* Corresponding Author

Ying-Ying Huang *

Mail address: Heng Hua Road 18, Hengyang, 421002, China.

Email: huangyy@hnit.edu.cn

Tel:+86-15622102957

Table S1 Statistics on the comparison results of each sample.

| Sample | Number of cells | Mean reads per cell | Total detected genes | Median UMI Counts per cell |
| --- | --- | --- | --- | --- |
| QLQ | 19,705 | 21,859 | 11,815 | 1,426 |
| T308 | 21,603 | 17,043 | 11,831 | 1,327 |

Table S2 Comparison of cluster-enriched genes to known marker genes and cell type determination of QLQ.

| Target_Cluster | Gene Name | Known marker match | score | cell type |
| --- | --- | --- | --- | --- |
| 0 | caa43 | AT2G38750 | 83.5 | Endodermis |
| 0 | Isoform0014842 | AT5G10280 | 72.8 | Endodermis |
| 11 | Isoform0017077 | AT2G26530 | 97.1 | Endodermis |
| 11 | At5g65660 | At5g65660 | 80.8 | Endodermis |
| 15 | At4g37250 | At4g37250 | 75.7 | Endodermis |
| 15 | At5g58300 | At5g58300 | 74.5 | Endodermis |
| 15 | AT2G28670 | AT2G28670 | 72.917 | Endodermis |
| 1 | EARLI1 | AT2G29070 | 75.1 | Epidermis |
| 1 | LOX1.5 | AT1G55020 | 74.4 | Epidermis |
| 5 | FLA8 | AT4G12730 | 82.3 | Epidermis |
| 5 | PCAP1 | AT4G20260 | 79.6 | Epidermis |
| 10 | Isoform0018785 | Os06g0194900 | 74.5 | Epidermis |
| 12 | Isoform0016860 | Os10g0536600 | 94.7 | Epidermis |
| 12 | PNC1 | AT3G05290 | 87.6 | Epidermis |
| 2 | PSBY | AT1G67740 | 88.3 | Cortex |
| 2 | Isoform0003244 | AT1G45130 | 82.9 | Cortex |
| 4 | AT2G23090 | AT2G23090 | 79.4 | Cortex |
| 4 | UCC1 | AT5G13180 | 76.9 | Cortex |
| 6 | Isoform0013868 | Os05g0363200 | 81.2 | Trichoblast |
| 6 | ACL5 | AT3G49960 | 74.9 | Trichoblast |
| 8 | Isoform0001452 | AT4G37640 | 74.1 | Trichoblast |
| 8 | AGP31 | AT1G28290 | 82.4 | Trichoblast |
| 7 | PER47 | AT1G70460 | 87.3 | Root Cap |
| 9 | GH3.1 | AT2G14960 | 85.7 | Root Cap |
| 9 | GRXC9 | AT5G63030 | 89.4 | Root Cap |
| 14 | AT5G59970 | AT5G59970 | 82.6 | Root Cap |
| 14 | AT2G37470 | AT2G37470 | 81.8 | Root Cap |
| 3 | PATL3 | AT1G72150 | 83.5 | sub-cell type of stele |
| 3 | Isoform0016448 | AT4G35350 | 74.9 | sub-cell type of stele |
| 13 | AT2G03500 | AT2G03500 | 78.4 | Phloem |
| 13 | Isoform0018927 | AT5G01600 | 82.5 | Phloem |
| 16 | Isoform0018012 | AT4G29100 | 97.1 | pericycle |
| 16 | KIN1 | AT5G15960 | 92.3 | pericycle |
| 17 | IRX12 | AT2G38080 | 85.1 | xylem |
| 17 | Isoform0016448 | AT4G35350 | 74.6 | xylem |
| 18 | AT4G34480 | AT4G34480 | 88.3 | xylem |

Table S3 Comparison of cluster-enriched genes to known marker genes and cell type determination of T308.

| Target_Cluster | Gene Name | Known marker match | score | cell type |
| --- | --- | --- | --- | --- |
| 0 | EARLI1 | AT2G29070 | 75.1 | Epidermis |
| 5 | PER4 | AT1G14540 | 83.1 | Epidermis |
| 5 | Isoform0015322 | AT3G25110 | 79.9 | Epidermis |
| 7 | Isoform0018785 | Os06g0194900 | 74.5 | Epidermis |
| 7 | Isoform0003223 | Os06g0194900 | 80.8 | Epidermis |
| 8 | WAT1 | AT1G75500 | 83.2 | Epidermis |
| 8 | Isoform0012771 | Os11g0247300 | 83.6 | Epidermis |
| 1 | Isoform0012469 | AT5G01210 | 81.2 | Trichoblast |
| 1 | Isoform0001452 | AT4G37640 | 74.1 | Trichoblast |
| 12 | CYP94A2 | AT3G48520 | 81.5 | Trichoblast |
| 12 | Isoform0009020 | AT2G21540 | 80.1 | Trichoblast |
| 2 | Isoform0021108 | Os03g0794700 | 82.6 | Endodermis |
| 4 | Isoform0019869 | Os04g0349500 | 79.6 | Endodermis |
| 4 | CBF5 | Os07g0636000 | 76.1 | Endodermis |
| 3 | Isoform0003244 | AT1G45130 | 82.9 | Cortex |
| 3 | Isoform0018335 | AT2G37180 | 78 | Cortex |
| 9 | VTC2 | AT4G26850 | 82.4 | Cortex |
| 9 | Isoform0021503 | Os02g0823100 | 78.3 | Cortex |
| 14 | Isoform0000689 | AT5G57110 | 74.4 | Cortex |
| 14 | AT2G23090 | AT2G23090 | 79.4 | Cortex |
| 6 | AT5G59970 | AT5G59970 | 82.6 | Root Cap |
| 6 | H2B-3 | AT2G37470 | 81.8 | Root Cap |
| 13 | Isoform0017890 | AT2G38750 | 97.3 | Root Cap |
| 13 | Isoform0019970 | AT2G26530 | 90.1 | Root Cap |
| 10 | ANN4 | AT2G38750 | 86.9 | sub-cell type of stele |
| 10 | Os01g0722300 | Os01g0722300 | 100 | sub-cell type of stele |
| 11 | Isoform0003745 | ATMG00520 | 94.4 | xylem |
| 11 | Isoform0018164 | AT4G01850 | 81.9 | xylem |
| 15 | WDL3 | AT3G04630 | 72.9 | procambium |
| 15 | Isoform0018009 | AT1G04550 | 87.9 | procambium |
| 16 | Isoform0008681 | AT2G03500 | 78.4 | Phloem |
| 16 | Isoform0018927 | AT5G01600 | 82.5 | Phloem |

Table S4 The primers used for qPCR validation.

| Gene name | Froward primer 5’-3’ | Revers primer 5’-3’ |
| --- | --- | --- |
| EARLI1 | TTTGCACCGCCATTAAAGCC | ACCATCCAAGCCATGAGAGC |
| FPF1 | CTCAACATGTCCGGCGTTTG | AGGTGAACCGTTGATCGCTT |
| At2g23090 | CCTAGCCTAGCCGACAAAGG | GCTTTGGGATGCTTTGCCTC |
| At2g28670 | TTGTGACCAATCCCGCTCTC | TTGCCGTTGTTCTGAATGGC |
| At4g35350 | CTTTCTCCAAGCTGCTGTGC | AGCTTCTCTTCCACGCTGTC |
| CYP94A2 | TTTCCAGGACATTCTCCGGC | CTGAACCTTCCACGTCGTCA |
| *Ia*Actin | CAGCACACTCCAACAGGTTT | GACCGGACTCATCATACTCTGC |
| PER72 | GAAAATCTGCTCGCGGGTTC | GGCACTCCTGGAGTCTTTCC |
| PIN1 | ATTACCCAGCTCCAAACGCA | AACAGGAGAGGCACTTGAGC |
| ARF8 | GCAGGTGAAAGGCAACCAAG | TCTGTGCCATCCAGGGAAAC |
| CYP7072A | CTTTCAATGTGGCGCTGCTT | ATTGTCGGCAATCTGGTCGT |
| LAC2 | AGCGCCTTTCGATAACACCA | GAGTCCCATTTGGTCCCTGG |
| FER2 | TTCATGGTGCCCATAACCCC | AAACTTGGCCAGCCCTTTGA |
| ZP1 | CCCGGGGCATACCTTAGTTG | AGCGTCCGAAGATGAAGCAA |
| ROP2 | CAACATGGACATTGGCTGGC | ACACTGCATTGACTGGGGAG |
| IAA17 | TCAAGAAAGTCCGCCGTCAA | TTCCTAAGGTATGGTGCGCC |
| EIN3 | ACCCCACGACATGAACATCC | GCCAAGCCTTTGAGCAGAAC |

Figure S1 Correlation plots between scRNA-seq pseudo-bulk expression and bulk RNA-seq expression of QLQ (A) and T308 (B) root tips under Cd stress.


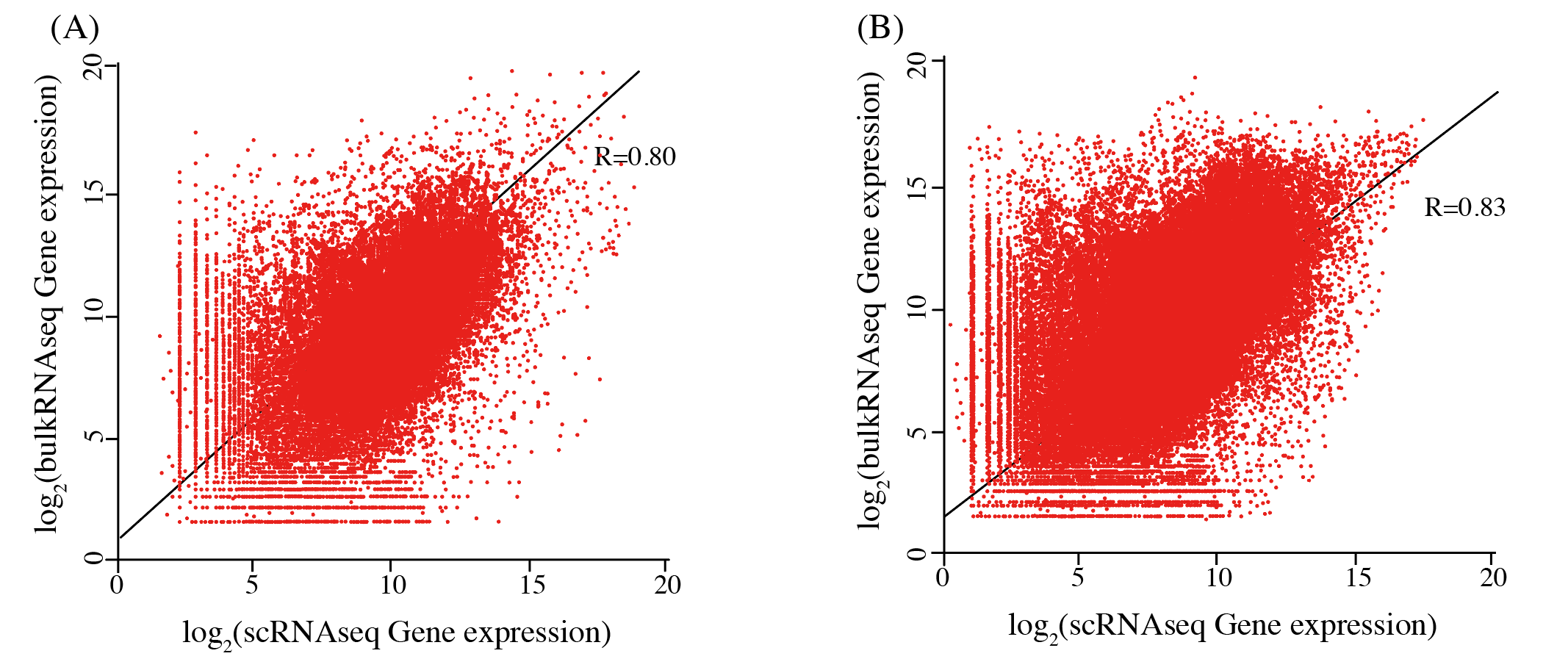


Figure S2. Isolation of epidermis, cortex and endodermis, vascular cylinder, and root hair tissues from water spinach root tips. (A) Epidermis isolated using a razor blade. (B) Vascular cylinder dissected using forceps. (C) Residual tissues after removing the vascular cylinder, representing the cortex and endodermis. (D) Isolation of trichoblast tissues.


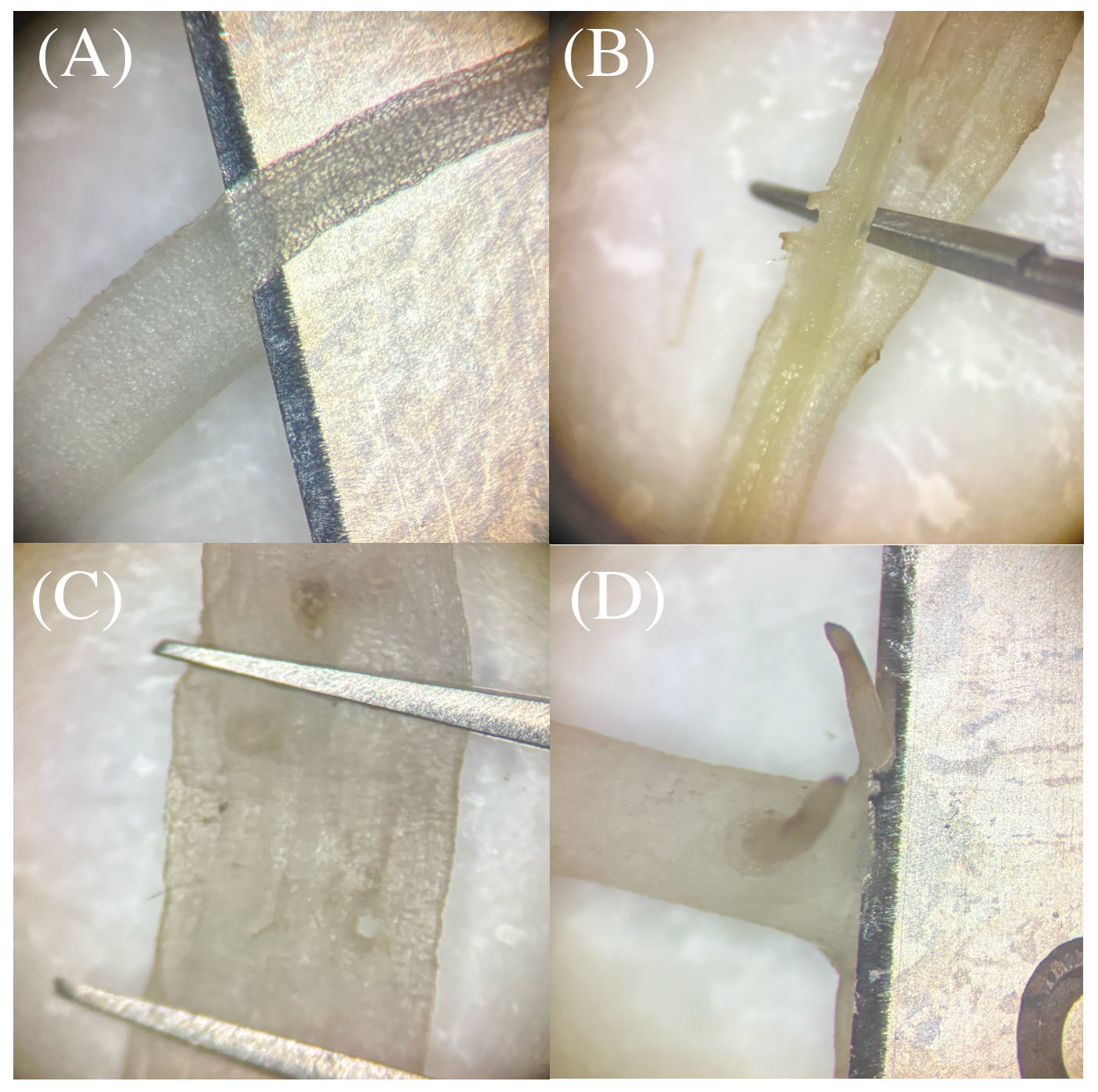


Figure S3 The functional analysis of DEGs in the same cell types between QLQ and T308. (A) The UpSet plot shows the DEGs in QLQ compared to T308 and the number of cell types carrying single DEGs or multiple DEG combinations. (B) GO enrichment analysis of DEGs in different cell types comparing QLQ to T308. Note: P-values were obtained using hyper-geometric test, with g:SCS (graph-based stratified Cox-Snell) correction applied for multiple comparisons.


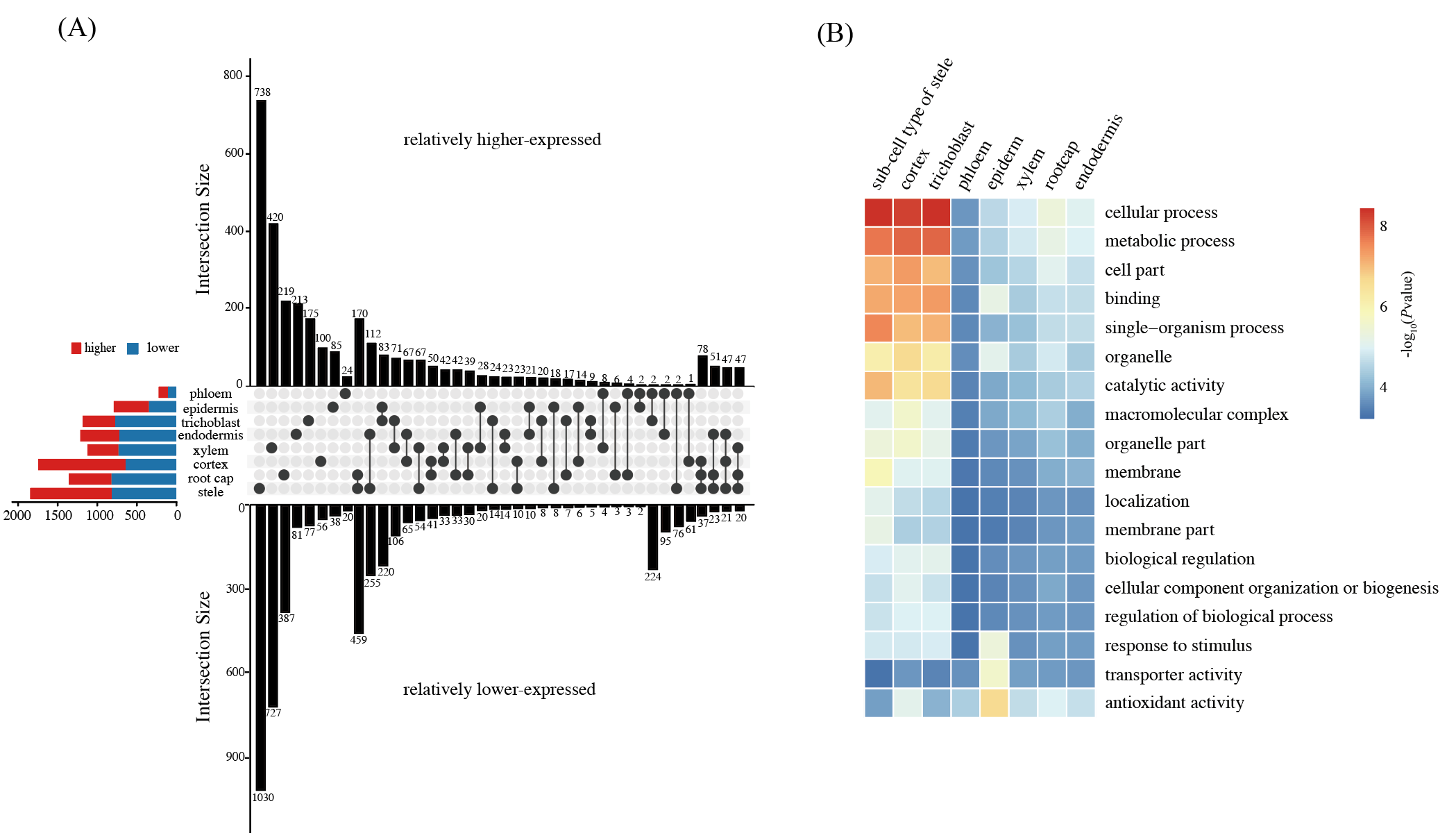

Supplement: Web_Material_uhaf077 [file web_material_uhaf077.zip › Web_Material_uhaf077.docx]
